# Experimental Assessment of Intestinal Damage in Controlled Donation After Circulatory Death for Visceral Transplantation

**DOI:** 10.3389/ti.2023.10803

**Published:** 2023-01-12

**Authors:** Pablo Stringa, Leandro Emmanuel Vecchio Dezillio, Paloma Talayero, Javier Serradilla, Agustina Errea, Mariana Machuca, Rodrigo Papa-Gobbi, Onys Camps Ortega, Melisa Pucci Molineris, Natalia Lausada, Ane Miren Andres Moreno, Martin Rumbo, Francisco Hernández Oliveros

**Affiliations:** ^1^ Transplant Group, La Paz University Hospital Health Research Institute (IdiPAZ), Madrid, Spain; ^2^ Department of Pediatric Surgery, La Paz University Hospital, Madrid, Spain; ^3^ Institute for Immunological and Pathophysiological Studies (IIFP), School of Exact Sciences, National University of La Plata, National Council of Scientific and Technical Research (CONICET), La Plata, Argentina; ^4^ Organ Transplant Laboratory, School of Medicine, National University of La Plata, La Plata, Argentina; ^5^ Immunology Department, 12 de Octubre University Hospital, Madrid, Spain; ^6^ Special Pathology Laboratory, Faculty of Veterinary Sciences, National University of La Plata, La Plata, Argentina; ^7^ Biochemistry Research Institute of La Plata, School of Medicine, National University of La Plata, National Council of Scientific and Technical Research (CONICET), La Plata, Argentina; ^8^ Executive Operational Committee, ERN TransplantChild, Madrid, Spain

**Keywords:** experimental transplantation, organ procurement, donation after cardiac death, solid organ transplant, intestinal transplantation

## Abstract

There is an urgent need to address the shortage of potential multivisceral grafts in order to reduce the average time in waiting list. Since donation after circulatory death (DCD) has been successfully employed for other solid organs, a thorough evaluation of the use of intestinal grafts from DCD is warranted. Here, we have generated a model of Maastricht III DCD in rodents, focusing on the viability of intestinal and multivisceral grafts at five (DCD5) and twenty (DCD20) minutes of cardiac arrest compared to living and brain death donors. DCD groups exhibited time-dependent damage. DCD20 generated substantial intestinal mucosal injury and decreased number of Goblet cells whereas grafts from DCD5 closely resemble those of brain death and living donors groups in terms intestinal morphology, expression of tight junction proteins and number of Paneth and Globet cells. Upon transplantation, intestines from DCD5 showed increased ischemia/reperfusion damage compared to living donor grafts, however mucosal integrity was recovered 48 h after transplantation. No differences in terms of graft rejection, gene expression and absorptive function between DCD5 and living donor were observed at 7 post-transplant days. Collectively, our results highlight DCD as a possible strategy to increase multivisceral donation and transplantation procedures.

## Graphical Abstract

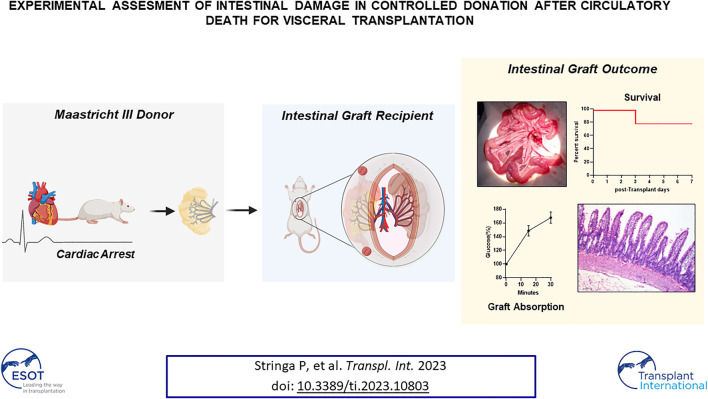

## Introduction

The universal shortage of organs has prompted the growing use of donations after circulatory death (DCD), which is expected to escalate further (1). Various organs including kidneys, liver, lungs, pancreas, and heart from DCD donors have been successfully employed for transplantation in several centers worldwide. The majority of the pitfalls and concerns regarding DCD donor use have been addressed by strict donor selection (2), improvements in normothermic regional perfusion (3), and *ex situ* machine perfusion devices (4). Although the actual impact of routine DCD use is challenging to assess, it has been estimated that organ donation has increased by 42% (5).

The valuable resource of DCD has been denied for intestinal grafts due to concerns regarding the ischemic susceptibility of this organ. Notably, experimental studies underwriting this veto are limited and underscored by heterogeneous methodology (6–8). Conversely, clinical evidence supports the use of these grafts in clinical settings. Moreover, there is an urgent need for grafts to cope with the mismatch between the waiting list for pediatric and adults intestinal transplantation (ITx) and scarcity of available organs. ITx candidates are predominantly those with longer waiting periods and higher mortality rates compared to other patients awaiting solid organ transplantation (9). Therefore, a thorough evaluation of the potential use of intestinal grafts from donors with DCD becomes highly relevant.

The aims of this study were therefore: 1) to develop an experimental model of Maastricht III DCD for solid organ transplantation in rodents, focusing on the viability of intestinal grafts; 2) to compare intestinal grafts quality from DCD to that in brain death donors (BDD) and living donors (LD) in the corresponding animal models and to assess the factibility of using intestinal graft from DCD in an experimental model of ITx; 3) to evaluate graft viability from DCD donors after ITx in a rat model.

## Materials and Methods

### Animals

Adult (8–10 weeks) male Sprague-Dawley and Wistar rats were used in this study. The animals were group-housed in a climate-controlled room on a 12-h light–dark cycle at the animal facilities of our institution. Experimental protocols were performed in strict accordance with the recommendations of the *European Union Criteria for Animal Use in Scientific Experimentation* (63/2010 EU) and related Spanish legislation (RD 53/2013). The experimental procedures were approved by the local *Animal Welfare Ethics Committee* (PROEX 58.7/20).

### Experimental Design

**FIGURE 1:**
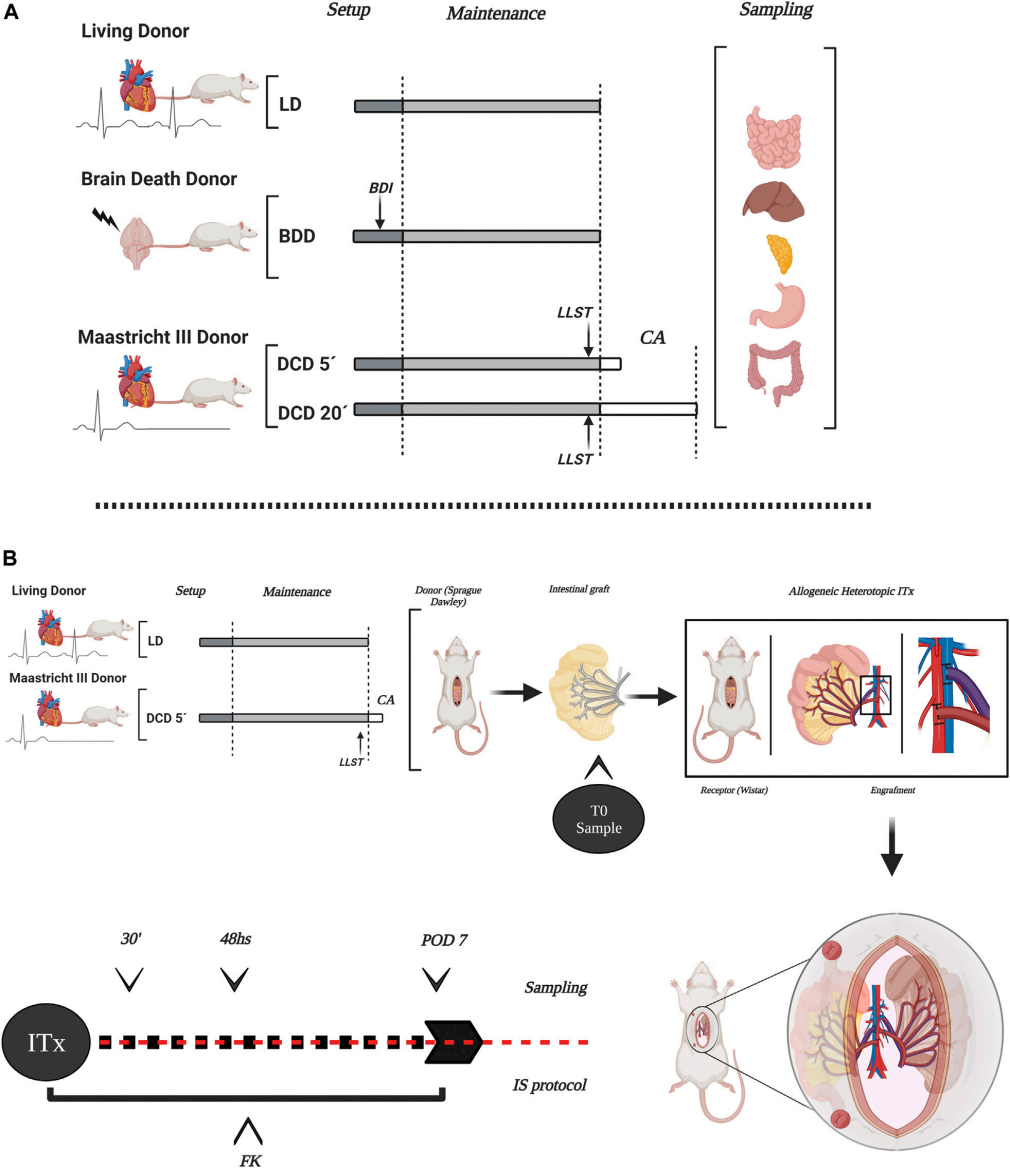
Scheme of the experimental design in the different donation models and ITx. The study encompassed several types of organ donors in rats, representing the different stages and component organs of intestinal and multivisceral grafts **(A)**. Experimental design of allogeneic-heterotopic ITx model in rats. DCD5 and LD were considered to ITx. Transplanted intestines were sampled after transplantation to evaluate long-term outcome of the graft **(B)** LD, living donors; LVD, ventilated living donors; BDD, brain death donors; BDI, brain death induction; DCD, donation after circulatory death with two different times of CA (5 and 20 min); LLST, limitation of life support therapy; CA, cardiac arrest. This figure was generated with BioRender program (biorender.com).

For the study of intestinal graft quality coming from different donation scenarios, animals were divided into five experimental groups (Five animals per group) (Figure 1A): 1. Conventional LD: i.v. organ perfusion; 2. Ventilated LD (VLD): mechanical ventilation for 2 h, i.v organ perfusion; 3. Donation after 5 min of circulatory death (DCD5): mechanical ventilation for 2 h, 5 min of CD, i.v. organ perfusion 4. Donation after 20 min of circulatory death (CD): mechanical ventilation for 2 h, 20 min of CD, i.v. organ perfusion (10); 5. Brain Death (BD): BD and ventilation for 2 h, i.v organ perfusion (11, 12). Additional information from experimental procedures is available in Supplementary Data S1.

In all groups, samples of the different organs comprising the multivisceral graft (small bowel, stomach, pancreas, colon, and liver) were taken for histological analysis (13–20).

For long-term intestinal graft survival evaluation after allogeneic and heterotopic ITx, two experimental groups was performed (Five ITx per group) (Figure 1B): 1- LD + ITx, and 2: DCD5 + ITx. Additional information is available in Supplementary Data S2.

Samples of transplanted intestine were taken through intestinal ostomy at 0, 30 min, 48 h and 7 days after ITx. Histological and morphometric graft evaluation, Immunofluorescence staining, gene expression analysis and graft functional evaluation was performed (21) (Supplementary Datas S3–S7).

## Results

### Feasibility of Controlled Maastricht III DCD in Rats

The experimental model developed in this study enabled the replication of crucial phases of Maastricht III DCD (patient maintenance, donor limitation of life support therapy (LLST), diagnosis of CD, hypoperfusion phase, waiting period after cardiac arrest (no-touch period), cannulation time, washing, and multivisceral graft retrieval). The success of the Maastricht III model in rats for visceral transplantation (VT) was 83.3%. Two animals presented complications during the procedure and were excluded from the study (one case of prolonged hypotension and one case of massive hemorrhage through the artery cannula).

**FIGURE 2:**
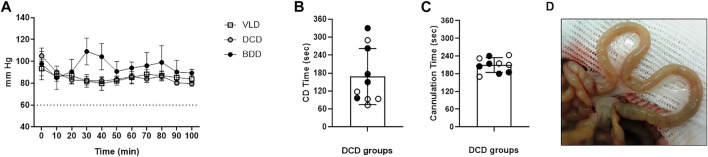
Donor and graft monitoring during DCD. **(A)** MAP in living donor (LD), brain death (BD), and donation after circulatory death (DCD) groups exhibited normal values throughout the experimental procedure. No significant differences were observed between groups at any time-points. **(B)** Elapsed period (seconds) between limitation of life support therapy and CA diagnosis in the DCD groups. **(C)** After “no-touch period” cannulation time was measured from skin incision to the start of cold perfusion (black and white circles correspond to donation after 5 min of circulatory death [DCD5] and donation after 20 min of circulatory death [DCD20] groups, respectively). **(D)** Representative macroscopic image of an intestine from a DCD20 donor at the end of the experimental procedure. The pale appearance denoted correct washing of the graft.

Successful donor monitoring was achieved in all experimental groups based on stable body temperature, O_2_ saturation, and mean arterial pressure, which were established as inclusion parameters as described in the Materials and Methods section. BDD exhibited a slight increase in mean arterial pressure (MAP) at 30 and 40 min after BD induction, which was attributed to the norepinephrine infusion that was concurrently administered to this group (Figure 2A).

The time elapsed between the LLST and diagnosis of CD was 2.8 ± 1.4 min. No significant differences were observed between DCD5 and DCD20 (Figure 2B). CD temporal heterogeneity was not correlated with MAP at the time of LLST (data not shown). After “no-touch period” (5 or 20 min, according to experimental group), time required for opening, cross-clamp, cannulation and perfusion was 3.4 ± 0.35 min in DCD5 and 3.46 ± 0.35 in DCD20 groups (No significant differences between groups) (Figure 2C). Considering hypoperfusion phase, waiting period after cardiac arrest and cannulation, total warm ischemia time was 8.41 ± 0.35 min and 23.5 ± 0.34 for DCD5 and DCD20, respectively (*p* < 0.05).

At the time of sampling, the macroscopic appearance of intestinal grafts showed pale appearance in the five experimental groups, without traces of blood in the mesenteric vasculature or small intestinal wall, indicative of correct washing of the graft even in DCD20 (Figure 2D).

### Morphological Characteristics of Multivisceral Grafts From DCD

**FIGURE 3:**
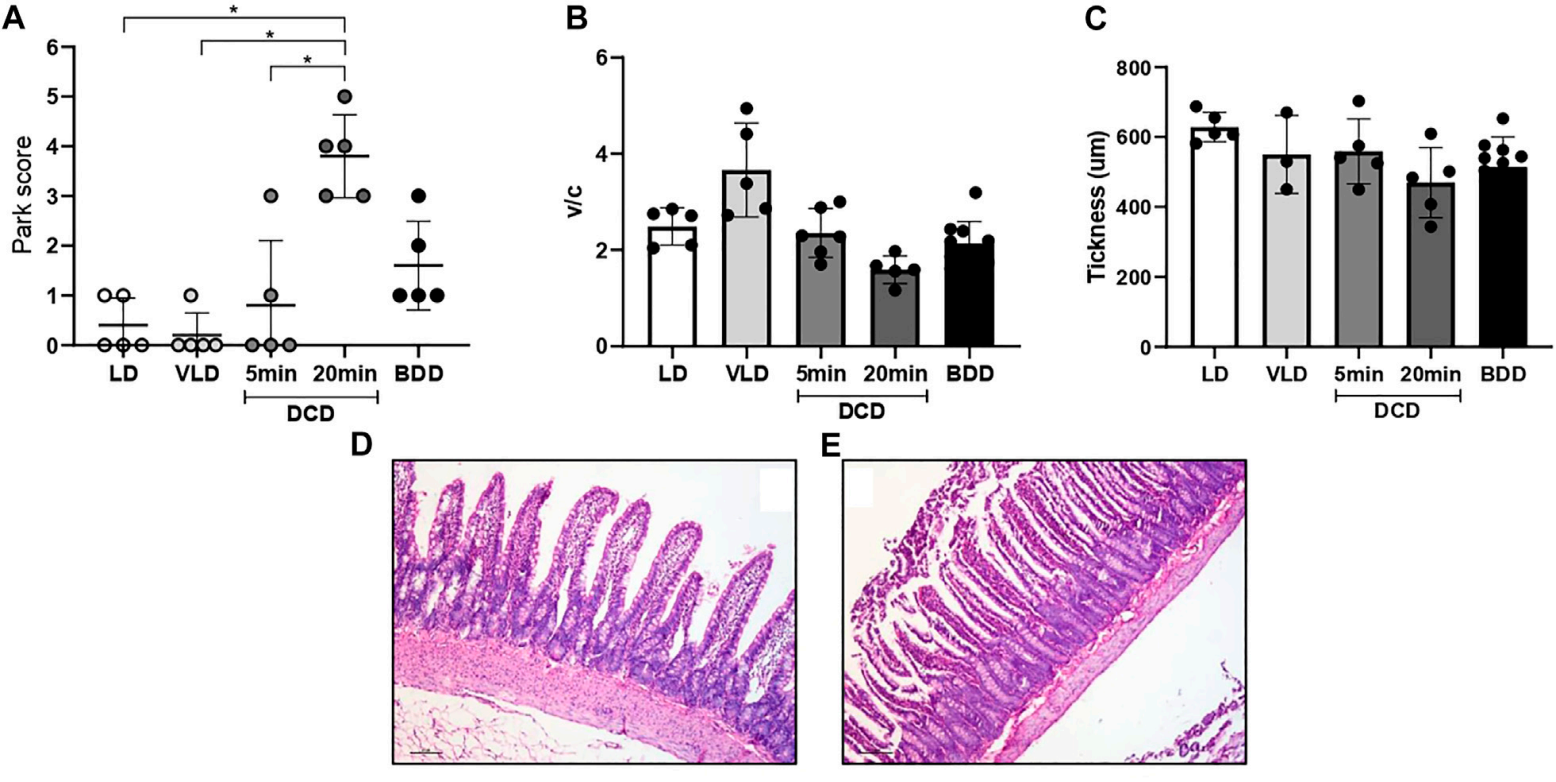
Warm ischemia time impacts intestinal graft histology in donation after circulatory death (DCD). **(A)** Architecture in DCD5-group small bowel was similar to that of LD. Groups with the longest ischemia time presented with more severe damage based on Chiu/Park scores compared to the other donors, with significant differences vs. LD and DCD5 groups (*p* < 0.05). **(B,C)** Despite a worsening trend in the crypt villus index and intestinal mucosa thickness in the donation after 20 min of no-touch period (DCD20) group, no differences between groups in these morphometric studies were observed. Representative microphotographs from DCD5 **(D)** and DCD20 **(E)** group.

**FIGURE 4:**
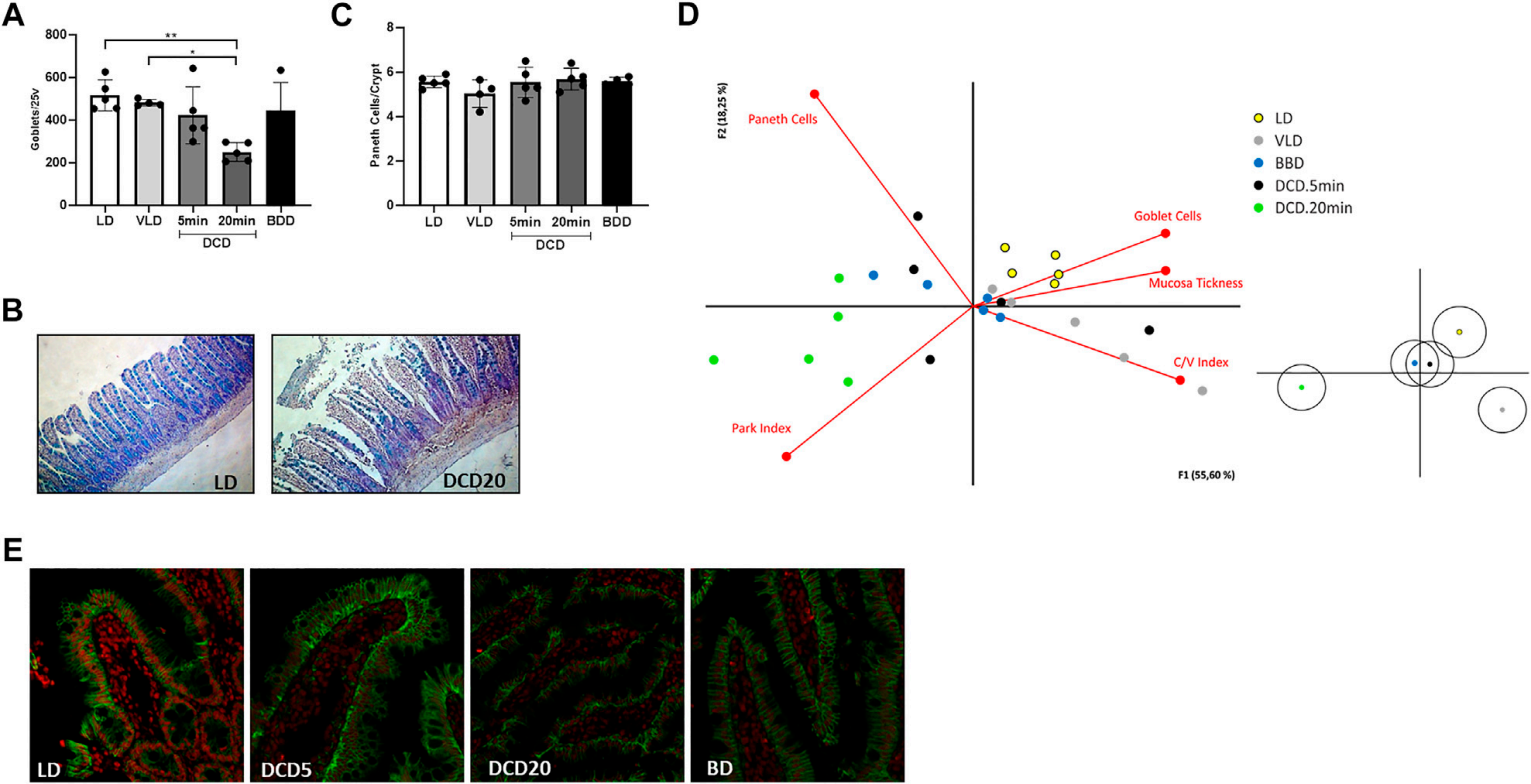
Cell population study and principal component analysis of the intestinal graft in the different donation scenarios. **(A)** Goblet cell count using Alcian Blue staining. Significant differences between DCD20 and LD groups were observed (*p < 0.05; **p < 0.01). **(B)** Examples of high and low numbers of goblet cells in intestinal samples corresponding to LD and DCD20 groups, respectively. **(C)** All experimental groups exhibited similar numbers of Paneth cells. **(D)** Biplot visualizing correlations among variables. The DCD5 group closely resembled BDD (grafts used in human settings). Samples from the DCD20 group were the most distinct compared to the other experimental groups and were associated with histological damage variables based on Chiu/Park scores. Claudin-3 staining in different scenarios of intestinal procurement **(E)**.

The DCD group exhibited time-dependent small bowel histological damage. Prolonged CD generated substantial intestinal mucosal injury (3–5 based on the Park/Chiu score). Significant differences were observed between the DCD20 versus LD and DCD5 groups (*p* < 0.05, Figure 3A). However, no significant differences were observed in villus/crypt index and mucosal thickness between experimental groups (Figures 3B,C). Similar results were observed in all groups with the exception of DCD20 small bowel samples, in which GC count was considerably lower. A significant difference in Goblet cell (GC) analysis was noted between DCD20 and LD groups (*p* < 0.05, Figures 4A,B). Conversely, no differences were observed in Paneth cells per crypt count, with an average of approximately 5 Paneth cells in all study groups (Figure 4C). DCD20 group showed major alterations in tight junction proteins ZO-1 and claudin-1 expression (Figure 4E), whereas minor alterations were observed in DCD5 and BD groups.

The average variability explained by principal component analysis was 73% (Figure 4D, principal component 1, 55%; principal component 2, 18%). GC number, mucosal thickness, and villus-crypt index were directly correlated with LD and VLD groups, suggesting that higher GC number, thicker mucosa, and increased crypt-villus index were indicative of small bowel integrity. Park/chiu score was inversely correlated with these variables but was strongly correlated with the DCD20 group. Of note, despite data dispersion, BDD and DCD5 groups were clustered closer to the VD and VLD groups than to the DCD20 group.

**FIGURE 5:**
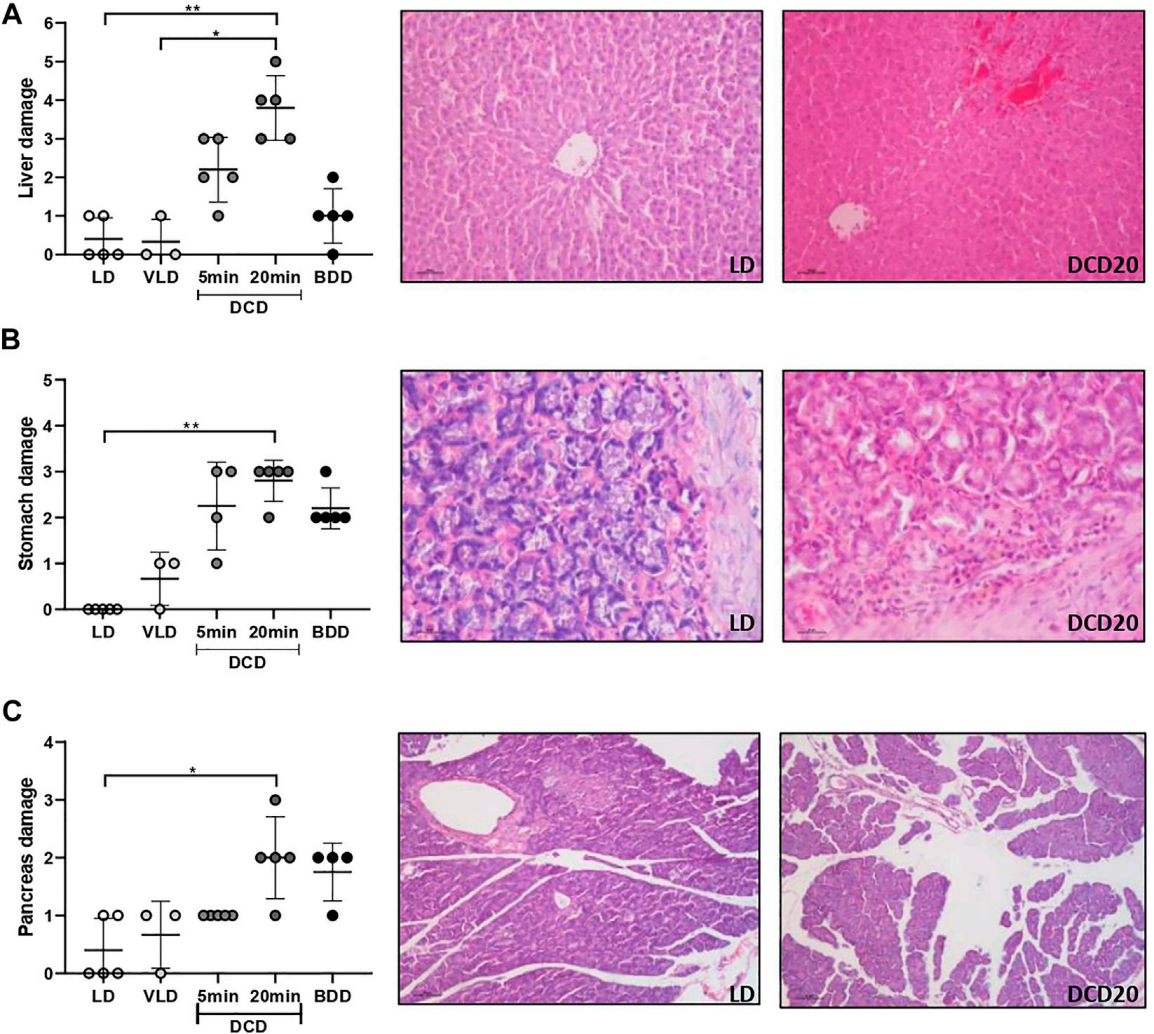
Histological analysis of the remaining organs comprising multivisceral grafts in the different donation scenarios. Liver **(A)**, stomach **(B)**, and pancreas **(C)** analyses are presented. In all cases, significant differences were observed between the donation after 20 min of CA (DCD20) and LD groups (*p < 0.05; **p < 0.01). Representative microscopic images of low and high damage of the three organs are presented.

In agreement with the trend observed for intestinal grafts, other organs were also affected by longer periods of DCD: DCD20 livers revealed significantly poorer morphology compared to LD (Figure 5A), with mild-to-moderate congestion and cytoplasmic vacuolization as the most relevant microscopic alterations (DCD20 vs. LD group, *p* < 0.05).

The DCD20 group presented more severe gastric injury, accompanied by glandular damage, epithelial desquamation, and cellular infiltrate of a mild-to-moderate degree (*p* < 0.05 vs. LD group). Similar but milder injuries were observed in DCD5 and BDD gruops (Figure 5B).

Moderate edema was the most common observation in the pancreas of BDD and DCD20 groups (Figure 5C). A significant difference between DCD20 and LD in pancreas histological damage was observed (*p* < 0.05).

Slight edema was observed in a subset of DCD and BDD colon samples. No alterations in this organ were detected in LD. No significant differences in the analysis on colon samples were observed between groups.

### Long-Term Outcome of Allografts From DCD was Similar to the Gold Standard (LD) After Experimental ITx

**FIGURE 6:**
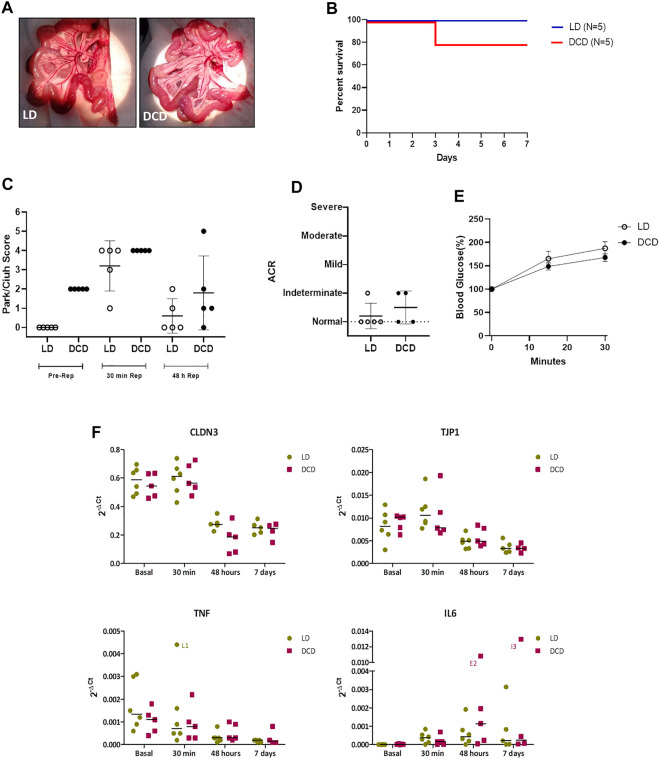
Intestinal Graft monitoring from DCD after ITx. Macroscopic appearance of transplanted intestines during intra-surgical graft reperfusion (DCD5 and LD) **(A)**. Survival analysis of DCD and LD ITx recipients **(B)**. Histological analysis of Ischemia-Reperfusion injury **(C)** and acute cellular rejection **(D)** of the DCD5 and LD graft after ITx did not show significant differences between groups. Intestinal graft absorption **(E)** and gene expression of CLDN3, TJP1, TNF and IL-6 after transplantation were similar in DCD5 and LD animals **(F)**.

Considering the results obtained in the graft characterization we decided to perform ITx using a heterotopic model previously described. In order to determine if DCD5 may affect the long-term transplant outcome, in spite of the lack of differences with LD graft. To this aim, two groups were subject to ITx using either DCD5 or LD grafts. Upon vascular anastomosis, DCD5 grafts reperfusion was fast and homogeneous, comparable to LD grafts (Figure 6A). Post-operative recipient recovery was appropriate and weight and clinical evolution was comparable in DCD5 and LD groups. Similar results were observed in survival curve (Figure 6B), only 1 animal from DCD group died in 3rd post-operative day, which was the same animal that showed extensive ischemia/reperfusion damage at 48 h post-transplant (Figure 6C). Anyway, no statistical significant differences in mortality were observed between groups. All animals survived showing no signs of graft rejection until 7 post-operative days, when the protocol was ended. During ITx, serial samples of graft were obtained to evaluate ischemia/reperfusion damage (Figure 6C). Interestingly, although DCD5 donors have a Park/chiu score 2 points higher than LD in the pre-reperfusion sample, showing the impact of CD on intestinal histology as shown in previous experiments. Upon engraftment, the highest damage was observed in the 30 min post-reperfusion sampling, where both DCD5 and LD grafts showed similar damage (median Park/chiu score of 4 in both groups). Recovery from damage was evident in the 48 h post-reperfusion sample of LD and DCD5 groups. Histopathological analysis was performed on intestinal graft samples taken at day 7 post-operative. No signs of rejection were observed in either LD or DCD5 groups (Figure 6D). Furthermore, functional capacity of grafts was assessed at day 7 post-ITx using a glucose absorption test. Either LD or DCD5 grafts showed appropriate absorptive function, indicating that DCD procedure has not impacted in absorptive capacity of the graft (Figure 6E).

To determine comparative effects of DCD and LD on additional pathways, gene expression analysis was performed in graft samples taken at different times: just before the dissection procedure (basal samples) and 30 min, 48 h and 7 days after ITx (Figure 6F). ZO1 and claudin-3 were analyzed as markers of epithelial integrity, and IL6 and TNFα as reflection of pro-inflammatory condition. According to what observed in the histological analysis, no differences were observed between LD and DCD in any of the genes and time-points studied (Figure 6F). Both groups showed a high expression of claudin-3 in basal and 30′ samples which decreased significantly after 48 h (LD: *p* = 0.002, DCD: *p* = 0.006), this correlating with the Chiu-Park score normalization in the biopsies. A similar but smoother dynamic was observed in zonulin expression, showing the LD group a significant increase at 30’ vs. basal samples (*p* = 0.010). Regarding the pro-inflammatory cytokines, TNFα showed the highest levels in basal samples, decreasing progressively afterwards. Nevertheless, IL6, which was almost undetectable at the beginning of the procedure, increased after reperfusion (Figure 6F). Overall, this experiment indicates that selection of appropriate DCD grafts results in a transplantation procedure with comparable outcomes to the LD experimental gold standard scenario.

## Discussion

Patients on solid organ transplantation waiting lists worldwide outnumber annual transplant procedures. Donor shortage is a main contributing factor in this regard, and the situation is expected to deteriorate unless clinical alternatives are identified (5, 22). DCD is a significant contributor to the donor pool in countries with legislation for DCD use. In some cases, DCD represents up to 40% of transplanted grafts. BD donation activity remains underdeveloped in several countries, and DCD is a valuable practical alternative for deceased organ donations (1, 23).

VT constitutes a notable challenge for DCD, as the liability of the intestinal barrier to ischemia and discouraging results from animal models have precluded its implementation in clinical practice (7). The availability of experimental models that recapitulate the main features of DCD will facilitate the development of novel interventions with promising results in preclinical settings (24). Consequently, our initial aim was to develop a model of experimental DCD that recapitulates the main features of the Maastricht III donation criteria.

In an interesting article, Søfteland et al have reported that rats and humans are more susceptible to intestinal ischemia-reperfusion injury than pigs, and laboratory animal characteristics must be considered in the experimental design of investigations. Following these conclusions, we consider that the selected specie to carry out the present work was adequate, and allows us to reliably study the impact of DCD procedure on multivisceral graft quality and ITx viability (25).

The experimental procedure of controlled DCD developed in the present work accurately reproduces the different phases of a Maastricht III donors used in the clinical practice. This model permits the characterization of distinct DCD-related features and provides a valuable tool for assessing different translational strategies to improve the quality of VT grafts. The maintenance period with mechanical ventilation and monitoring of vital parameters such as temperature and MAP in the DCD model constitute one of the strengths of the present work. In addition, the diagnosis of CD, the 5 min of no-touch period and cannulation maneuver before graft washing was based on the clinical practice of pediatric Maastricht III donors, highlighting the relevance of this model.

Our results demonstrated that the histopathological features of the small bowel from DCD with less warm ischemia time were similar to those of grafts from BDD in terms of Chiu/Park score, crypt-villus index, and intestinal mucosa thickness. We emphasize the good quality of the architecture of intestines from donors with CD, given the relatively short period of cardiac arrest prior to washing and organ removal. Roskott et al. reported that elevated expression of heat shock proteins occurs in the human intestine after DCD and BDD grafts exhibited higher IL6 expression levels compared with those in DCD (6). These data suggest that grafts for visceral transplantation warrant different pre-treatments in accordance with their immunological profiles. In this regard, our model unlocks a range of possibilities for evaluation in preclinical and clinical settings.

We observed greater histological damage in the small intestine than in colon samples, confirming the importance of studying the small bowel barrier as a more sensitive parameter of ischemic damage. This finding is in accordance with a previous study by Bresler et al. which described similar differences in ischemic damage between the small bowel and colon in an experimental model (20). With regard to specific cell populations, GC number was significantly lower in the experimental group with more severe histopathological damage (DCD20) but was similar between DCD5 and BDD. These results are consistent with previous reports about the impact of ischemic damage on GC (14, 25). Although Paneth cells exhibit differential sensitivity to Ischemia/reperfusion injury, we observed that warm ischemia in both DCD groups resulted in minor changes in the Paneth cell population, which exhibited cell counts similar to those in BDD (16, 26).

Principal component analysis revealed that the intestines of the DCD5 and BDD groups were similar, whereas the DCD20 small bowel differed from that of the other experimental groups, which constitute encouraging results regarding intestinal graft quality. In addition to the intestine, visceral grafts may be composed of other organs, as previously stated. In our work, we assessed the impact of different types of donations on the stomach, pancreas, and liver. As shown in Figure 5, ischemic time negatively impacted donors with CD who experienced prolonged warm ischemic time. Notably, similarities in histological damage in the small intestine were noted between DCD5 and BDD groups. Considering these encouraging results, we also showed that DCD5 intestines can be engrafted using a small bowel rat heterotopic model with comparable results to LD intestine (Figure 6).

Decision to compare DCD-derived intestinal grafts with a no-touch waiting time of 5 min (similar to the clinical scenario of pediatric transplantation) with LD ITx procedures is based on two justifications: as reported by Oltean, in the experimental field of ITx in rodents, all published work where transplantation is performed use LD. Also, we considered that the great challenge for DCD was to compare it with the “gold-standard” donor situation regarding solid organ transplantation (27).

Notably similar rejection-free graft survival and graft function were observed in both groups and although a trend in increased ischemia-reperfusion damage was observed in DCD grafts, overall performance was similar, showing the feasibility of the use of DCD graft intestines at least in the condition tested. In agreement with reported warm ischemia period in solid organ transplantation and previous experimental experiences, we have selected a short warm ischemia time that allowed intestine to be transplanted without major damage. Cobianchi et al. have shown that using a pig small bowel transplantation model, having 20 min of cardiac arrest before DCD generates an intestinal damage that afterwards severely affects ITx outcome. In the rat model used, we observed that 20 min of cardiac arrest induce important changes in intestinal mucosa. However, having shorter periods allow the obtention of intestinal grafts that can be used in an ITx procedure without affecting the outcome. Current clinical practice has shifted towards normothermic perfusion in DCD donors with superior results to those achieved with traditional cold perfusion which is similar to the perfusion employed in the present work (28). Therefore, future work should implement the necessary devices to establish normothermic perfusion in this model.

In summary, the present work provides a detailed description of a novel experimental model of Maastricht III DCD that may contribute to evaluation of the feasibility of DCD for VT in the clinical practice. Our results suggest that the quality of visceral grafts from DCD and short ischemia times closely resemble those of BDD with comparable performance and preservation of functional capacity in a heterotopic intestinal transplant model, highlighting DCD as a possible solution to the clinical imbalance between the demand for organ donation and transplantation procedures.

## Data Availability

The raw data supporting the conclusion of this article will be made available by the authors, without undue reservation.

## Abbreviations

BD, brain death; BDD, brain death donor; CA, cardiac arrest; CD, circulatory death; DCD, donations after circulatory death; DCD5, donation after 5 min of circulatory death; DCD20, donation after 20 min of circulatory death; GC, Goblet cell; ITx, intestinal transplantation; LD, conventional living donor; LLST, limitation of life support therapy; VLD, ventilated living donor; VT, visceral transplantation.
