# Supplementary material for: Experimental Assessment of Intestinal Damage in Controlled Donation After Circulatory Death for Visceral Transplantation
Source: Transpl Int. 2023 Jan 12;36:10803. doi: 10.3389/ti.2023.10803 (PMC9878676; doi:10.3389/ti.2023.10803)
Supplement: Supplementary file 1 [file DataSheet1.docx]

**SUPPORTING INFORMATION**

**S1 *Experimental Model of Maastricht III DCD***

Rats were anesthetized with isoflurane inhalation anesthesia (5% and 2–3% for induction and maintenance, respectively). Animals were placed in the dorsal *decubitus* position on a thermal blanket to enable insertion of a cannula in the right carotid artery connected to a multi-parametric DYNE MCO-300-07 monitor for blood pressure control (normal values: 70–140 mmHg). Subsequently, a tracheotomy was performed, and animals were ventilated for 2 h (2.5, 55/70 RPM, PEEP 0; Harvard Apparatus Rodent Ventilator model 683). Pulse oximetry (CMS-VESD, Multifunctional Visual Stethoscope, CONTEC) in the donor's leg was used to measure O_2_ saturation (typically between 95–100%) and pulse rate (typically > 250 bpm). Rectal temperature was registered (37.5–38ºC). All parameters were recorded every 10 minutes throughout the procedure.

After 2 h of ventilation, pneumothorax was induced via a diaphragmatic incision, as previously reported ^(10)^. To avoid a dramatic drop in MAP, artificial sartificial ventilation was continued for 5 min in donor rats with induced pneumothorax. After these 5 minutes, mechanic ventilation and thermal support were not provided and LLST was performed, leading to pre-death hypoperfusion and warm ischemia period time. Based on the parameters of pediatric donation, death was diagnosed by absence of pulse on invasive arterial blood pressure monitoring and absence of spontaneous breathing. Different circulatory death (CD) times were evaluated according to experimental groups (Figure 2-B). In order to reproduce the clinical situation a “no-touch” waiting period after CA (5 or 20 minutes depending on experimental group as showed in Figure 1-A) was considered. After stipulated CA time, opening, cross-clamp, abdominal aorta cannulation and perfusion was performed. Animals received 50 UI of heparin at time of LLST.

In order to compare the quality of grafts from different types of donors, a brain death (BD) group was included based on a previously reported model ^(11,12)^. Briefly, a 4F balloon cannula was placed subdurally via skull trepanation and insufflated at a flow rate of 1 mL/h. When the Cushing reflex was observed, an apnea test was performed to confirm BD. Animals in the BD group were anesthetized and monitored as described above for the CD group. As previously reported by our group and others, norepinephrine was used to prevent hypotension in the donor ^(12)^ .

In all experimental groups, organs were washed at the end of the protocol (Figure 1-A) with 40 mL of cold preservation solution solution perfused for 3 min through the arterial cannula using a perfusion pump (PC11-UBT, APEMA).  Finally, samples of multivisceral graft were taken (Figure 1-A).

**S2** ***Intestinal Transplantation procedure***

The heterotopic allogeneic (SD to Wistar) ITx procedures were performed as previously described. Briefly, the animals were anesthetized by isoflurane inhalation. Each graft consisted of entire small bowel. Celsior was used as preservation solution and cold ischemia time after graft retrieval was 60 minutes in all ITx procedures. The superior mesenteric artery and the portal vein of the graft were anastomosed to the infrarenal aorta and infrarenal cava vein, respectively.

Recipient received Tacrolimus daily (0.6 mg/Kg S.C) from the time of transplant until the end of experimental protocol (Figure 1-B)

**S3 Histological and morphometric analysis**

**Ischemic Damage**

Specific validated scores for the small bowel, stomach, pancreas, liver, and colon to quantify DCD and BDD-related damage were used. Intestinal hematoxylin-eosin samples were evaluated using the Chiu/Park score for ischemia-reperfusion injury: 0, normal mucosa; 1, subepithelial space at villus tip; 2, more extended subepithelial space; 3, epithelial lifting along villus side; 4, denuded villi; 5, loss of villus tissue; 6, crypt layer infarction; 7, transmucosal infarction; and 8, transmural infarction ^(13)^ . Intestinal villus height, crypt depth, and crypt thickness were assessed using FIJI software. At least 20 individual villi and crypts were measured per rat for each parameter. For the evaluation of goblet cells, Alcian blue staining with neutral red contrast was performed, and 25 villi per subject were quantified ^(14,15)^. Paneth cells were quantified in 20 longitudinal crypts per sample in representative microscopic fields (40 x), as previously reported ^(16)^.

Seven parameters were considered for each analyzed liver sample: cell necrosis, eosinophilic changes in hepatocytes, discohesive hepatocytes, cytoplasmic vacuolization, and sinusoidal dilatation. Each parameter was scored as follows: 0, normal; 1, mild change; 2, moderate change; and 3, severe change. Each sample was designated a general score based on the addition of each evaluated parameter ^(17)^.

Gastric evaluation was based on epithelial desquamation, mucosal hemorrhage, glandular damage, and eosinophilic infiltration, and was scored using a scale ranging from 0 to 3 (0, none; 1, mild; 2, moderate; and 3, severe) for each criterion ^(18)^. Edema, fat necrosis, and acinar necrosis were considered indicators of ischemic damage for pancreas analysis. Six grades of severity were used to quantify each parameter, as previously reported ^(19)^. A grading score for colon injury in rats reported by Bresler et al. was used. Briefly, the scale included edema, number of goblet cells, and mucosal alterations. Damage was scored from 0: no damage (Repleted Goblets cells and mucosa in contact with the muscular layer), to 4: Advanced mucosal injury (mucosal dissolution, epithelial breackdown) ^(20)^.

**Histological evaluation of Acute Cellular Rejection**

As we previously reported for graft rejection after experimental ITx, samples of the transplanted intestines obtained on post-surgical day 7 were evaluated following the criteria of Wu et al ^(21)^. Presence and severity of Inflammatory infiltrate, crypt epithelial injury, apoptosis, architectural distortion and mucosal ulceration was considered for determined absence, indeterminate, mild, moderate or severe grades of intestinal acute cellular rejection.

**S4 Immunofluorescence staining**

Sections of 5μm of small intestine tissue paraffin-embedded were deparaffinized and rehydrated according to standar procedures. Antigen retrieval was performed by heat treatment in citrate buffer( pH:6) and EDTA buffer (pH:8) for claudin-3 and ZO-1 respectively. Tissues samples were blocked using goat serum in PBS for 60 minutes at room temperature and then incubated overnight at 4°C with primary antibodies for ZO-1 (ThermoFisher, Cat#33–9100; 1:100) and claudin 3 (Abcam, Cat# ab15102; 1:100). After incubation samples were washed and incubated with secondary antibodies: Goat polyclonal Antibody to Mouse IgG ( H&L)- AlexaFluor 488 (Abcam, Cat# ab150113; 1:300) and Goat anti-Rabbit IgG (H + L) - AlexaFluor 488 (Life Technology, cat A11008; 1:300). Nuclear staining was performed with Methyl Green (1/400 from 2 % p/v stock) added into Fluorescent Mounting Medium (Dako, cat# S3023). Images were obtained with a Leica TCS SP5 Confocal Microscope and analyzed by the FIJI software.

***S5 Relative gene expression analysis***

Intestinal biopsies were submerged in RNAlater solution (Invitrogen, MA, USA) and stored at 4oC for 24h and then at -80oC until their processing. RNA was isolated with RNeasy Mini Kit (Qiagen, Hilden, Germany) and retrotranscribed to cDNA with High-Capacity cDNA Reverse Transcription Kit (Applied Biosystems, MA, USA).

The expression of Tjp1, Cldn3, Il6 and Tnfa genes was measured with predesigned TaqMan™ Gene Expression assays (Rn02116071_s1, Rn00581751_s1, Rn01410330_m1, Rn99999017_m1) using TaqMan™ Fast Advanced Master Mix on a 7500 Fast Real-Time PCR System (Applied Biosystems, MA, USA) according to manufacturer’s instructions. Threshold cycle (Ct) scores were calculated as the mean of the duplicates, and they were normalized against Ct scores of the endogenous control GAPDH (Rn01462661_g1). Relative expression was determined as 2-ΔCt, where ΔCt = Ct gene of interest – Ct endogenous control (Nat Protoc. 2008;3(6):1101-8. doi: 10.1038/nprot.2008.73).

***S6 Graft functional Evaluation***

The absorptive function of transplanted intestines was evaluated as previously described. Glucose 40% solution at a dose of 2 g/Kg was administered into the lumen of transplanted intestines. Peripheral glycemia was measured using an Accu-Chek blood glucose meter (Roche) just before, 15 and 30 minutes after glucose administration.

**S7 Statistical analysis**

Nonparametric methods were used for statistical comparisons. Statistical differences between groups were assessed using the Kruskal-Wallis test corrected for multiple comparisons using

Dunn’s test. Statistical analyses were performed using GraphPad software (version 8.00; San Diego, CA, USA). Statistical significance was set at p < 0.05

For gene expression analysis, differences between groups were assessed by Mann Whitney test, whereas paired t-test was used for comparing intragroup samples at different time points. P values <0.05 were considered statistically significant and GraphPad Prism (v 8.0.2) software was used for all the tests.

Biplot principal component analysis was performed to investigate the relationships between Chiu/Park score, mucosal thickness, Paneth and goblet cell number, and crypt-villus index, and their associations in different experimental groups.
